# Detecting Multiple Damages in UHPFRC Beams through Modal Curvature Analysis

**DOI:** 10.3390/s24030971

**Published:** 2024-02-02

**Authors:** Fahime Sokhangou, Luca Sorelli, Luc Chouinard, Pampa Dey, David Conciatori

**Affiliations:** 1 Water and Civil Engineering Department, Laval University, Quebec City, QC G1V 0A6, Canada; fahime.sokhangou.1@ulaval.ca (F.S.); luca.sorelli@gci.ulaval.ca (L.S.); pampa.dey@gci.ulaval.ca (P.D.); david.conciatori@insa-strasbourg.fr (D.C.); 2 Department of Civil Engineering, McGill University, Montreal, QC H3A 0G4, Canada; 3 ICUBE, UMR 7357, CNRS, INSA de Strasbourg, Université de Strasbourg, 67000 Strasbourg, France

**Keywords:** structural health monitoring, concrete beams, crack detection, vibration, curvature of mode shapes, crack, ultra-high-performance fiber-reinforced concrete

## Abstract

Curvature-based damage detection has been previously applied to identify damage in concrete structures, but little attention has been given to the capacity of this method to identify distributed damage in multiple damage zones. This study aims to apply for the first time an enhanced existing method based on modal curvature analysis combined with wavelet transform curvature (WTC) to identify zones and highlight the damage zones of a beam made of ultra-high-performance fiber-reinforced concrete (UHPFRC), a construction material that is emerging worldwide for its outstanding performance and durability. First, three beams with a 2 m span of UHPFRC material were cast, and damaged zones were created by sawing. A reference beam without cracks was also cast. The free vibration responses were measured by 12 accelerometers and calculated by operational modal analysis. Moreover, for the sake of comparison, a finite element model (FEM) was also applied to two identical beams to generate numerical acceleration without noise. Second, the modal curvature was calculated for different modes for both experimental and FEM-simulated acceleration after applying cubic spline interpolation. Finally, two damage identification methods were considered: (i) the damage index (DI), based on averaging the quadratic difference of the local curvature with respect to the reference beam, and (ii) the WTC method, applied to the quadratic difference of the local curvature with respect the reference beam. The results indicate that the developed coupled modal curvature WTC method can better identify the damaged zones of UHPFRC beams.

## 1. Introduction

As a significant proportion (~40%) of bridges in Canada built during the 1950s and 1960s are nearing the end of their anticipated service life, there is a critical need to accurately assess their condition and monitor the progression of damage [1]. Recent bridge collapses, such as the Concorde overpass [2], the Morandi Bridge in Italy [3] and the FIU Pedestrian Bridge in the United States [4], underscore the importance of improving the ability to predict significant structural deficiencies in bridges. For instance, before its sudden collapse, the I-W35 Bridge in Minneapolis was designated “structurally deficient” by the federal government primarily due to extensive corrosion in its bearings. Moreover, approximately 75,000 other bridges in the United States were similarly classified in 2007 [5]. Advancing Structural Health Monitoring (SHM) is crucial for efficiently managing infrastructures in contemporary bridge system management [6,7].

Among the available global methods for SHM, vibration-based testing methods are the most common for assessing the structural integrity of various constructions [8,9] through changes in modal frequencies, damping and mode shapes [10]. The analysis procedures include, among others, the frequency shift method, modal strain energy-based method, curvature-based method, flexibility-based method and frequency response curvature method [11,12]. Although the frequency shift is a straightforward and popular indicator of damage for level 1 SHM investigations, Farrar et al. [13] demonstrated that the frequency shift could not detect damage in the I-40 bridge over the Rio Grande. Daei et al. [14] proposed a two-stage method to solve the inverse damage identification problem in numerical models of beams and frames. In the first stage, damage locations were determined using the modal residual force vector (RFV) concept. In the second stage, they used an optimization method called the gravitational search algorithm (GSA) to estimate the severity of the damage. However, the method lacks robustness in the presence of noise and was not applied to experimental data.

Pandey et al. [15] first proposed mode shape curvature as a method to identify and locate damage in beams. Furthermore, they qualitatively observed that changes in the curvature of mode shapes increase with the severity of damage. Further applications of curvature-based methods have been shown to be effective in multiple 1D and 2D investigations for small-scale localized damage [16,17,18,19]. Typically, curvature-based damage detection techniques involve comparing the curvature of mode shapes obtained from the undamaged and damaged states of a structure. Various mathematical and statistical algorithms can be applied to quantify and analyze the disparities in curvature [17,20,21,22]. Among them, we can refer to the works of Wahab and Roeck [23], who applied the “curvature damage factor” to localize damage on the Z24 bridge in Switzerland, and Ratcliffe [24], who presented an index (modified Laplace operator, MLO) based on the comparison between the modal curvatures of the damaged mode shapes and a third-order polynomial (representing the undamaged local curvature).

Despite the widespread use of mode shape curvature in damage detection, these methods have a significant drawback: they are sensitive to noise due to the second-order differentiation of mode shapes. This differentiation has the potential to amplify even minor noise present in a mode shape. Wavelets are also used to detect discontinuities in signals and have been shown to be able to detect small defects in the presence of noise. Wavelet transforms (WT) can be applied to space-defined signals such as mode shapes [25] or the derivatives of mode shapes [26]. Solís et al. [27] proposed a damage detection method that utilizes a wavelet-based approach for the damage detection of steel beams. This method involves analyzing and summing the coefficients obtained through continuous wavelet transform applied to the difference between mode shapes in undamaged and damaged states. The addition process incorporates the utilization of the natural frequency to weigh the disparities in mode shapes. The resulting wavelet coefficients for individual mode shapes, along with the sum across all mode shapes, serve as parameters for damage detection. Cao et al. [28] formulated a new concept of complex-wavelet mode curvature to remove the noise and detect single and multiple damages in an aluminum beam.

Cosoli et al. [29] employed the modal curvature-based damage index and continuous wavelet transform (CWT) for damage identification in cement-based beams subjected to loading from bending tests. Following each loading level, an impact test was performed to assess the behavior of the concrete elements. A modal analysis was conducted. Mode shape curvatures were analyzed, and discontinuities were objectively identified through various processing techniques, including CWT.

Our work extends the application of enhanced SHM methods to new UHPFRC structures, which are increasingly being favored for their superior durability and compressive strength, but they have not been extensively studied for damage detection [30,31]. Notably, UHPFRC often totally or partially replaces steel bars with short steel microfibers, leading to diffused damage zones characterized by numerous fine cracks rather than a few localized sizeable structural cracks. Another challenge in applying SHM methods to these new materials is the non-uniform distribution of fibers and stiffness introduced during casting operations [32,33]. This research also addresses the issue of damage detection in the presence of distributed damage over different zones along a beam. The occurrence of multiple damage zones along a beam affects mode shapes and curvatures differently as a function of their relative location and can impact the diagnostic value of curvature-based methods. New damage identification indices have been proposed that improve diagnostics in the presence of multiple zones of damage by combining damage indices from multiple modes [34,35,36]. The proposed methods are extensions of curvature-based damage identification methods [29] that combine modal curvature methods with spatial smoothing procedures to mitigate the effect of noise from measurements. Noise is magnified when calculating the second derivative of mode shapes, which reduces the effectiveness of defect detection methods. Two noise reduction methods are proposed. The first is based on averaging damage indices derived from the first three modes of vibration, and the second is based on applying a wavelet transform to the squared absolute differences of curvature for each mode. The proposed methods are demonstrated with a finite element model of a typical beam with a range of spatially distributed defects and with experimental measurements on a set of undamaged and damaged beams. The results demonstrate that the proposed methods are effective for detecting and locating multiple damage zones in UHPFRC beams.

## 2. Materials and Method

### 2.1. Experimental Set-Up

#### 2.1.1. Materials

**Figure 1:**
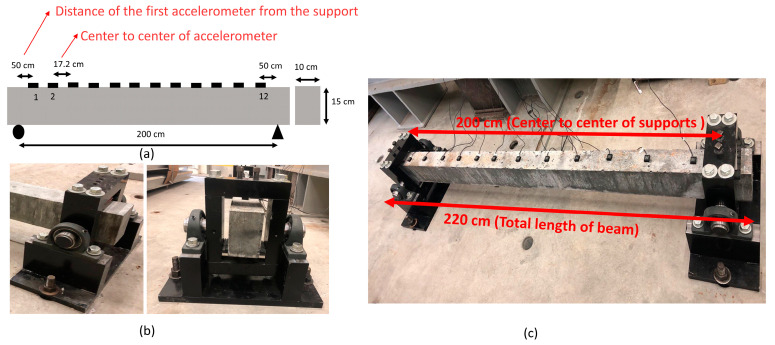
(**a**) Accelerometer position; (**b**) boundary condition details; (**c**) setup of beam.

The experimental beam was made of UHPFRC with a 2% steel fiber content and had a length (L) of 2 m and a rectangular section of 0.1 m by 0.15 m (Table 1). The beam was equipped with 12 evenly spaced accelerometers with roller-pin supports [37] (Figure 1). The Ductal concrete used in the fabrication of the beams has high compressive strength, ductility, longevity, eco-efficiency, insulation and aesthetic properties and is widely used in bridges, roads and innovative architectural designs [38]. The mechanical properties of the UHPFRC utilized in the experimental beams, along with the properties of the steel fibers, are presented in Table 2.

**Figure 2:**
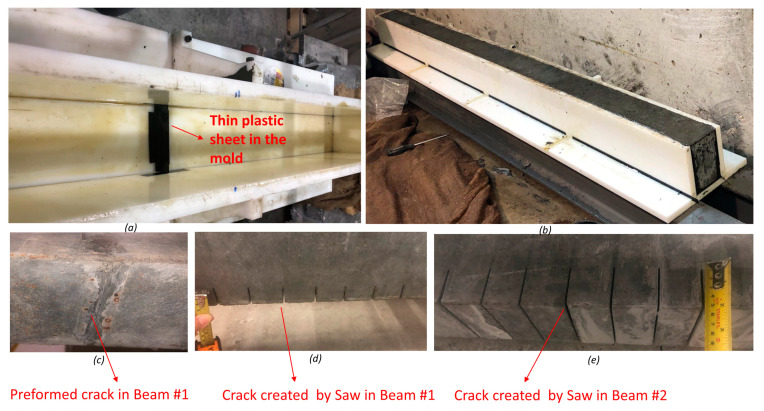
(**a**) Placement of sheet in the mold for preformed crack; (**b**) casting of concrete; (**c**) preformed crack; (**d**) simulated cracks for beam #1; (**e**) simulated cracks for beam #2.

Figure 2 illustrates the casting details, simulated cracks, beam setup and hinge boundary conditions. The beam was fastened to a metal box at each end with 6 bolts (3 at the top and 3 at the bottom), and the contact was adjusted using a 1 mm thin neoprene pad at the top and the bottom. Three beams were cast with the same mix and mold, ensuring similar mechanical properties and dimensions. One beam served as a reference, and cracks were intentionally introduced in the central zone (i.e., bending cracks) and at the end zone (i.e., shear cracks) for the other two beams. All cracks in the beams were created using a 2 mm saw, except for the shear crack in beam #1, which was preformed using a 1 mm plastic sheet.

#### 2.1.2. Vibration Test Setup and Instrumentation

For vibrational measurements, twelve accelerometers (Kistler, Mississauga, ON, Canada, model number: 8315A010B0AC06) were installed along the top surface of each beam with hot glue with spacing of 172 mm (Figure 1a), and they were connected to a Vishay Precision Group model 6100 data acquisition system. The accelerometers were single-axis sensors with an acceleration range of ±10 g, sensitivity of 200 mV/g, resonance frequency of 2000 Hz and mass equal to 15 g. A non-instrumented hammer (weight = 300 g) was used to provide input excitations at different locations along the top surface of the beam.

### 2.2. Finite Element Model

**Figure 3:**
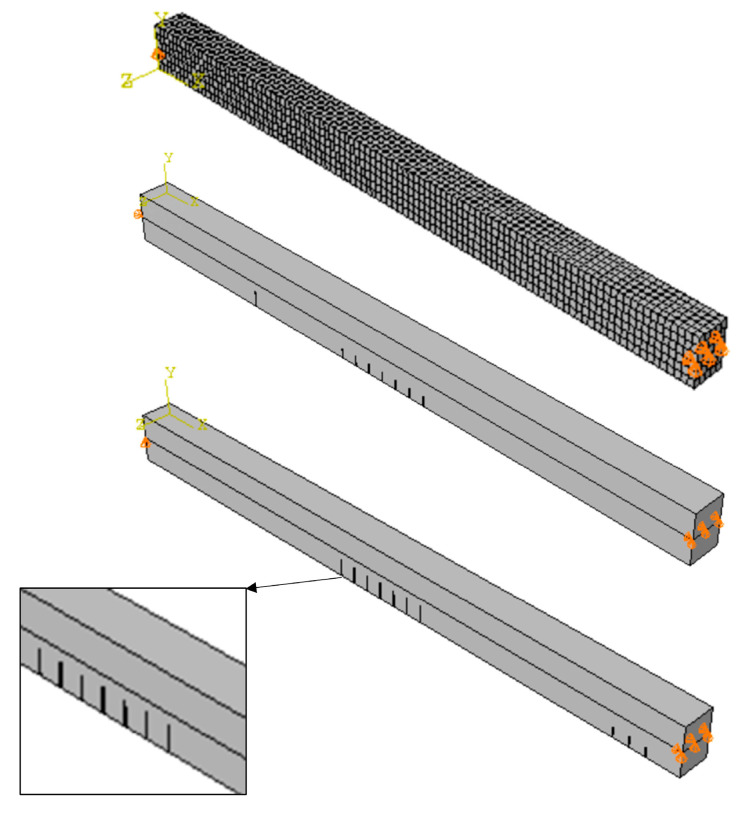
Mesh and crack modeling in the finite element model.

The Abaqus software 2020 was used to establish an FEM of the beam to assess the reliability of the experimental measurements and analysis procedures. The finite element (FE) model consisted of 2574 eight-node brick elements, as depicted in Figure 3. To visualize the mode shapes, the response of the middle points on the top side of the beam was considered. The configuration involved constraining the middle line of each end section of the beam to act as a hinge, simulating a pin joint. In practical terms, this implies that the beam was allowed to rotate freely at these designated points while still being able to transmit forces and moments across the joint. In the Abaqus computational model, the presence of cracks was explicitly defined as section discontinuities at the same locations as those in the experimental beams.

### 2.3. Damage Characterization Methodology

The proposed method for damage identification and localization in UHPFRC is an extension of the moment curvature method initially proposed by Pandey et al. [15]. First, the mode shapes of the beam are estimated from measurements with replicates to reduce the effects of experimental noise. Mode shapes and frequencies can be determined either by Experimental Modal Analysis (EMA) or Operational Modal Analysis (OMA) [39,40].

There are many modal identification methods for OMA data; among these, the Peak-Picking method (PP), the Enhanced Frequency Domain Decomposition method (EFDD), the Covariance-driven Stochastic Subspace Identification method (SSI-Cov), the poly-reference Least Squares Complex Frequency Domain method (p-LSCF) and the Automated Frequency Domain Decomposition (AFDD) methods are the most common [41]. A detailed description of each identification method is provided in the work of Raieneri et al. [42]. This work employs AFDD, a hybrid approach that merges the Frequency Domain Decomposition method with the Peak-Picking algorithm to extract mode shapes, eigenfrequencies and modal damping ratios from acceleration data gathered through ambient noise recordings.

Next, cubic spline smoothing is used to reduce noise in the estimated mode shapes before calculating mode shape slopes, curvatures and the mode shape curvature index (DIN). Cubic spline functions are very useful tools for smoothing noisy data. This is primarily because they offer a good tradeoff between simplicity and efficiency in controlling the degree of smoothing. MATLAB 2018 (MathWorks, Natick, MA, USA) was utilized as the primary computational tool for implementing the cubic spline smoothing method.

After applying the cubic spline smoothing method to the extracted mode shape, the modal curvatures are estimated. For beam-like structures, the modal curvature is defined as the second-order derivative of the mode shape function [15]:

(1) $$\frac{d^{2}\phi (x)}{dx^{2}}=-\frac{M(x)}{EI(x)}$$

where M(x), ϕ(x) and EI(x) denote the bending moment, mode shape and stiffness along the location of the beam (*x*), respectively. The equation indicates that local changes in stiffness due to damage result in corresponding changes in the mode shape slope and curvature. In this case, central differences are used to estimate the second-order derivatives of the mode shapes:

(2) $$\phi^{\prime \prime }=\frac{d^{2}\phi (x)}{dx^{2}}\approx \frac{\phi (x-h)-2\phi (x)+\phi (x+h)}{h^{2}}$$

where h is the distance between observations. In a previous work [43], Ho et al., defined the damage index of each *n*-mode by the squared absolute differences in mode shape curvature between the damaged beam (with *d* subscript) and reference states (with *r* subscript) as follows: $\left|\left({\left|\phi_{d,i}^{\prime \prime }\right|}^{2}-{\left|\phi_{r,i}^{\prime \prime }\right|}^{2}\right)\right|$, for each *i*-position along the beam. For normalization and facilitating a comparison of results across different modes, the normalized damage index $DI_{i}^{n}$ for each *n*-mode was normalized as follows:

(3) $$DI_{i}^{n}\overset{def}{=}\frac{\left|\left({\left|\phi_{n,d,i}^{\prime \prime }\right|}^{2}-{\left|\phi_{n,\;r,i}^{\prime \prime }\right|}^{2}\right)\right|}{\max(\left|{\left|\phi_{n,\;d,i}^{\prime \prime }\right|}^{2}-{\left|\phi_{n,\;r,i}^{\prime \prime }\right|}^{2})\right|}$$

where the denominator is defined as the squared absolute differences of each mode. In practice, even with replicates and spline smoothing, residual noise is present in experimental mode shapes, which can be amplified by curvature calculations that may be misinterpreted as damage or mask damage features. To further address this issue, the average of normalized damage indices for multiple n-modes is proposed as follows and serves as the first approach of this paper in reducing the noise:

(4) $$DIN_{i}=\frac{1}{N}\sum_{n=1..N}^{\;}DI_{i}^{n}$$

The normalized index for multiple modes $DIN_{i}$ is then a positive number between 0 and 1. A higher value of $DIN_{i}$ can be interpreted as the most likely damage locations. The first three modes were considered in this application.

#### Continuous Wavelet Transform (CWT) and Wavelet Transform Curvature (WTC)

The CWT of a signal f(x) is defined as [44]

(5) $$W(u,s)=\int_{-\infty }^{\infty }f(x)\frac{1}{\sqrt{s}}\psi (\frac{x-u}{s})dx$$

where ψ(x) is the wavelet kernel function, *s* is the scaling parameter and *u* is the shifting parameter.

The Mexican hat wavelet is the normalized second derivative of a Gaussian function g(x) [45,46]:

(6) $$g_{u,s}(x)=\frac{1}{\sqrt{2\pi \sigma }}e^{-(x^{2}/2\sigma )}$$

The Mexican hat wavelet exhibits better localization properties in both time and frequency domains compared to the Gaussian wavelet. It can effectively capture transient features or sudden changes in signals. The wavelet mother function of the Mexican hat can be derived as

(7) $$\psi_{M}(x)=\frac{d^{2}g(x)}{dx^{2}}=\frac{2}{\sqrt{3\sqrt{\pi }}}(1-x^{2})e^{-(x^{2}/2)}$$

where σ is the standard deviation of the Gaussian function and *x* is location along the beam. This wavelet is used to convolute f(x) as

(8) $$f_{u,s}^{*}=f⊗g_{u,s}^{\prime \prime }$$

Based on the characterization of the convolution operator,

(9) $$f_{u,s}^{*}=f⊗g_{u,s}^{\prime \prime }=f^{\prime \prime }⊗g_{u,s}$$

Equation (9) defines the wavelet transform curvature of the function f(x). In contrast to the differences in the mode shape curvature presented as a one-dimensional curve, the wavelet transform curvature provides a two-dimensional surface as a function of the location (*u*) and shape (*s*) parameters. The wavelet transform curvature with a progressively increasing shape parameter is effective in removing high-frequency noise components [17]. An alternative and complementary damage index can be proposed by calculating the WTC of mode shape curvature differences by applying a Mexican hat mother wavelet to the squared curvature differences (numerator of Equation (3)), which was shown to improve damage detection first by reducing the effect of noise and second by accounting for the jerk (third derivative of the displacement) and snap (fourth derivative of the displacement) of the mode shapes [47]:

(10) $$WTC(s,u)=\frac{1}{\sqrt{s}}\int_{-\infty }^{\infty }f(x)\psi_{M}(\frac{x-u}{s})dx$$

where f(x) is the squared absolute difference of the curvature of mode shapes and $\psi_{M}(.)$ is the Mexican hat mother wavelet.

## 3. Results and Discussion

The proposed method is initially demonstrated through its application to a beam modeled by finite elements, followed by its application to a set of experimental beams.

### 3.1. Strain Energy Density by FEM Static Analysis

**Figure 4:**
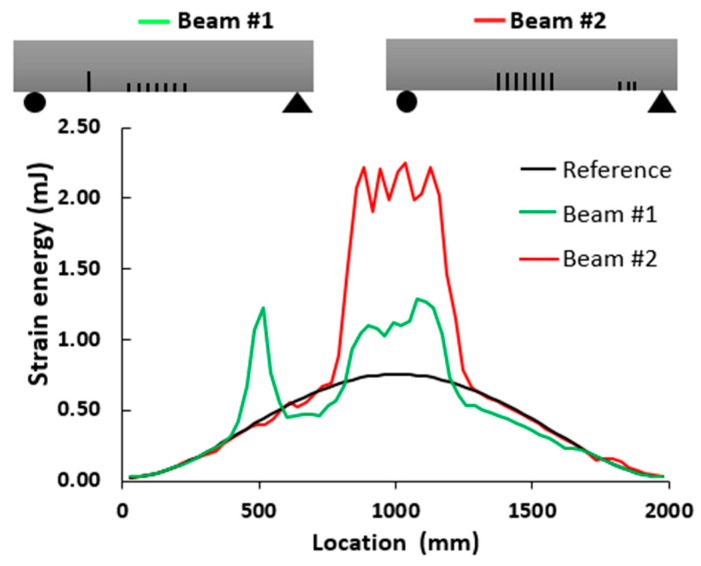
Total strain energy in different locations along the beams.

The objective of this section is to evaluate the distribution of strain energy within the beams through finite element static analysis. This assessment serves as a distinctive indicator of the damage distribution resulting from multiple cracks. The mesh size and element are presented in Section 2.2. Figure 4 illustrates the average magnitude of the strain energy in the elements located on the top surface of the reference beam, as well as two other beams exhibiting multiple damage locations.

In the case of beam #1, a substantial increase in strain energy was observed at the location of a single crack within the shear zone, as well as in the regions surrounding the middle of the beam. For beam #2, there was a noticeable increase in the strain energy in the middle section; however, the shear cracks did not have a significant impact on the strain energy. Moreover, the total strain energies for the reference beam, beam #1 and beam #2 were 13.57 mJ, 19.04 mJ and 25.47 mJ, respectively. This suggests an estimated increase in damage and deformability of 40% for beam #1 and 81% for beam #2. Although this study does not delve into the intensity of damage, it indicates that beam #2 was likely more damaged than beam #1.

### 3.2. Damage Detection Applied to the FEM Results

The FEM analysis results were obtained using a mesh of 20 mm with 120 elements on the top surface of the beam. For the modal curvature analysis, 33 equally spaced points (~60 mm) along the top surface of the beams were selected to represent the mode shape and then were smoothed before calculating the curvature. When comparing FEM results to experimental data, it is important to note that FEM benefits from a larger number of measurement points, reducing the impact of noise and bypassing limitations stemming from the available accelerometers.

**Figure 5:**
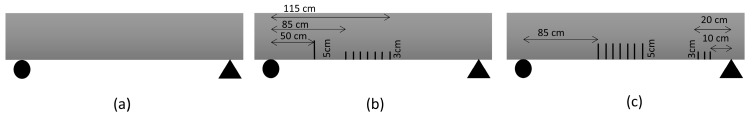
Damage scenarios for the FE beam model; (**a**) reference beam without crack; (**b**) beam #1 with bending cracks; (**c**) beam #2 with shear cracks and bending cracks.

Whereas the reference beam had no cracks, beam #1 had seven cracks (3 cm depth and 5 cm spacing) in its mid-section to simulate distributed bending damage and one single crack 50 cm from the left end support. Beam #2 had seven cracks (5 cm depth and 5 cm spacing) in its mid-section to simulate distributed bending damage and three cracks (5 cm depth and 5 cm spacing) in the end section of the beams to simulate distributed shear damage (Figure 5).

**Figure 6:**
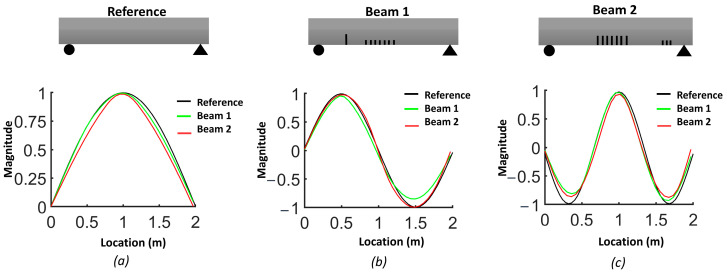
Comparison of the normalized mode shapes for the three beams: (**a**) first mode; (**b**) second mode; (**c**) third mode.

**Figure 7:**
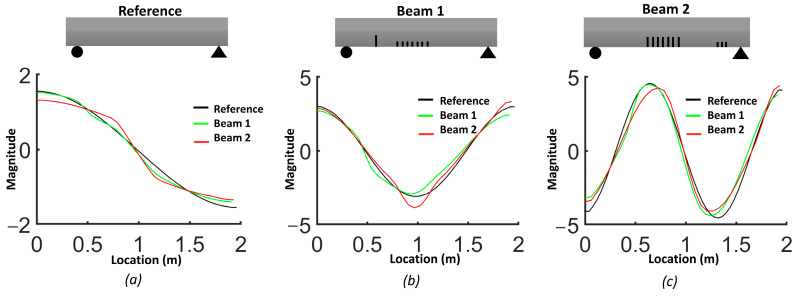
Comparison of the slope of mode shapes for the three beams: (**a**) first mode; (**b**) second mode; (**c**) third mode.

**Figure 8:**
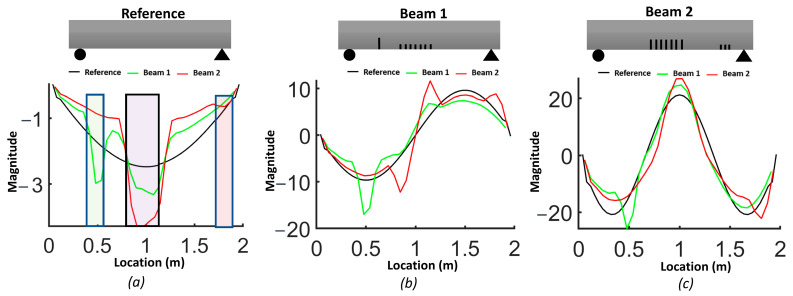
Comparison of the curvature of mode shapes for the three beams: (**a**) first mode; (**b**) second mode; (**c**) third mode.

The natural frequencies from the FEM are provided for each beam in Table 3. The results demonstrate how the natural frequency and mode shapes changed in response to the level and location of damage. The position of the distributed cracks was not initially identifiable from the first three mode shapes (Figure 6). However, the slopes and curvatures of the mode shapes significantly enhanced the identification of regions with cracks (Figure 7 and Figure 8). For beam #1, the first and third frequencies had the highest change (15.6% and 11.2%, respectively), as expected, since the cracks in the middle affected the first and third frequencies. Beam #2 exhibited higher changes in the first and third frequencies and lower changes for the second frequency due to the single crack with a 5 cm depth, which had more influence than three cracks with a 3 cm depth.

Although the first three mode shapes had shifts in their shapes, they did not provide information on the position of damage. Figure 7 shows that only the first derivative of the second mode provided information on damage in the middle of the beam and that the slopes from other modes failed to detect damage. Figure 8a illustrates that the second derivative of the first mode can be a robust indicator for cracks in both the middle and shear zone. The shaded areas represent the zones where cracks were located and highlight the regions where there were significant differences in curvatures. Figure 8b shows that cracks in the shear zone of the beam can affect the second derivative of the second mode. The curvature of the third mode can detect the presence of damage, similar to the curvature of the first mode, but the latter is more effective in locating damage.

**Figure 9:**
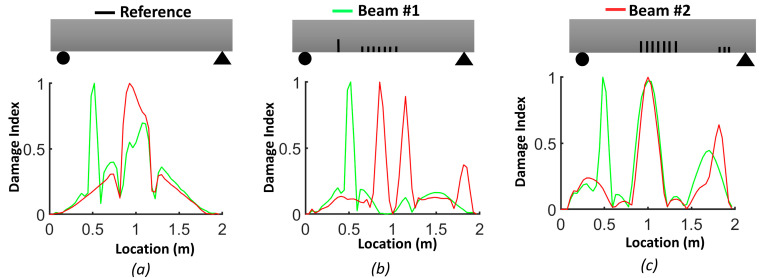
Damage index (*DI*) for FEM beams: (**a**) mode 1 (*DI*^1^); (**b**) mode 2 (*DI*^2^); (**c**) mode 3 (*DI*^3^).

**Figure 10:**
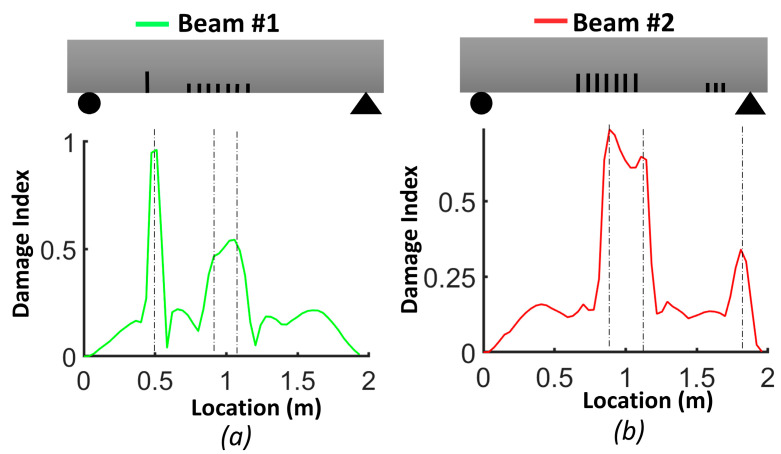
Damage index (*DIN*) for FEM beams: (**a**) beam #1; (**b**) beam #2.

Figure 9 illustrates the damage index (*DI*, Equation (3)) for the numerical models of beams #1 and #2. The damage index for the first mode (*DI*^1^) correctly identified the location of the two levels of distributed bending cracks, as well as the isolated crack located 50 cm from the left support of beam #1. *DI*^2^ based on mode 2 was more robust for detecting the single crack in beam #1 and the three cracks in the shear zone of beam #2. The damage index based on the third mode (*DI*^3^) had a larger value for all cracks in beam #1 and beam #2. In general, we can conclude that the damage index based on the third mode was better for detecting and localizing the damage. Figure 10 demonstrates the *DIN* plot for beam #1 and beam #2, with dashed lines indicating the crack positions in the numerical models.

In the case of beam #1, the *DIN* plot indicates that the isolated single crack had the highest value and was located 51 cm from the left support. There were two other dashed lines: one at 91.2 cm and another at 105.8 cm from the left support. For beam #2, in the middle of the plot, two dashed lines were positioned at 88.6 cm and 110.8 cm from the left support. Additionally, there was a dashed line representing the shear zone, situated 181 cm from the left support.

### 3.3. Damage Detection Applied to Experimental Results

The sampling frequency was set to 10 kHz, and each series of measurements was replicated at least 20 times. The AFDD procedure was used on each set of measurements to derive the auto-spectral and cross-spectral matrices of the acceleration records. The ranked singular values of the spectral matrix (PSD) were calculated, and the resonant peaks were selected as bending mode frequencies. For each beam, the average value and standard deviation of the frequencies and damping ratio for the first five natural frequencies are presented in Table 4 and Table 5. In the case of the first frequencies, the standard deviations were close to zero, indicating minimal variation or dispersion among the results. The variability of frequencies increased for higher modes of bending. Regarding the damping ratio, the low standard deviation indicates that the measured damping ratios were very close to each other, implying a high level of precision in the measurements.

**Figure 11:**
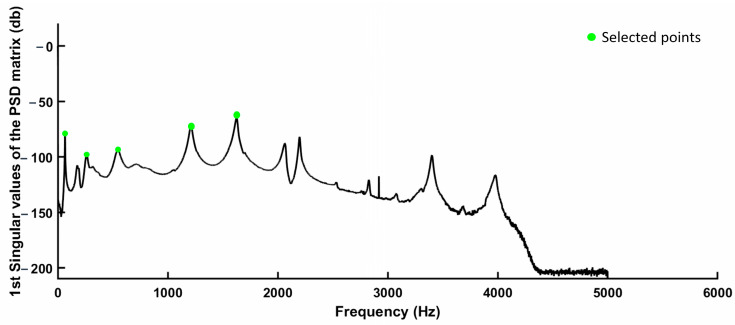
Example of the first singular values of the PSD matrix and selecting bending mode frequencies.

Figure 11 illustrates the selection of singular values from the power spectrum density (PSD) matrix, with green points representing the modal bending frequencies. It is important to note that the peak situated between the first and second bending peaks corresponds to a torsional mode and was not selected.

**Figure 12:**
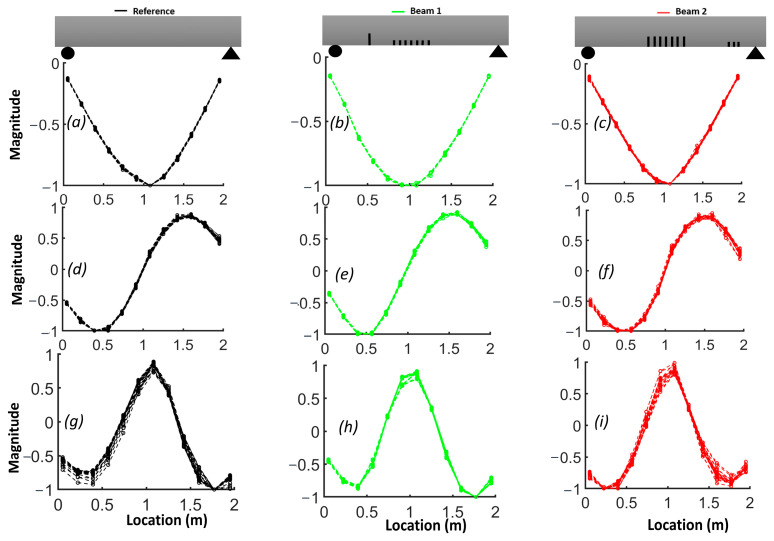
Replicates of (**a**–**c**) first mode shapes, (**d**–**f**) second mode shapes and (**g**–**I**) third mode shapes for reference beam, beam #1 and beam #2.

The plots of the first three mode shapes before smoothing are shown in Figure 12 with 20 replicates for each beam. Upon observing these plots for three different beams, it can be concluded that the second and third modes exhibited more variability than the first mode.

**Figure 13:**
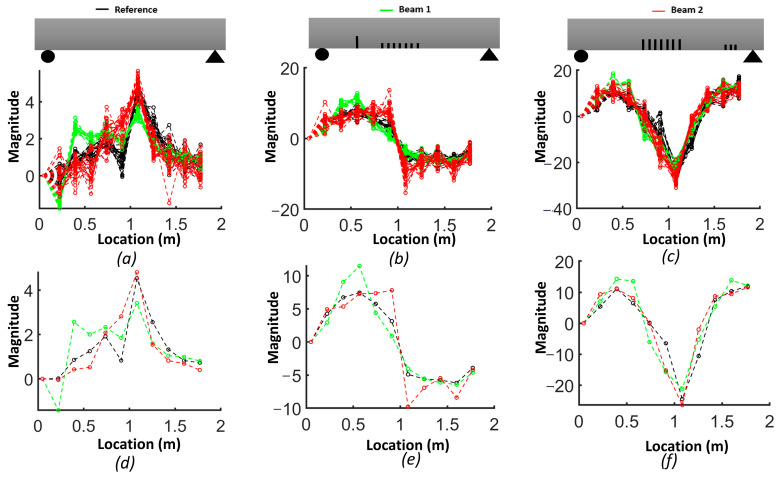
Curvature of bending mode shapes: (**a**) mode 1; (**b**) mode 2; (**c**) mode 3; (**d**) average for mode 1; (**e**) average for mode 2; (**f**) average for mode 3.

Figure 13 displays the curvature of mode shapes calculated directly from the measurements obtained from the 12 accelerometers. Concerning the reference beam, the results for the curvature of the first mode show that the curvature can show apparent anomalies that could be associated with the non-uniform distribution of the steel fibers of UHPFRC in a zone of high curvature. Indeed, the fiber distribution is rarely uniform in a UHPFRC beam due to casting methods, the wall effect and segregation [48,49,50]. Interestingly, such an effect was not visible for the second and third mode of the reference beam at the same location. The variability in the properties of the beams is a complication factor for damage identification because the employed method relies on the reference beam to define the damage zone.

**Figure 14:**
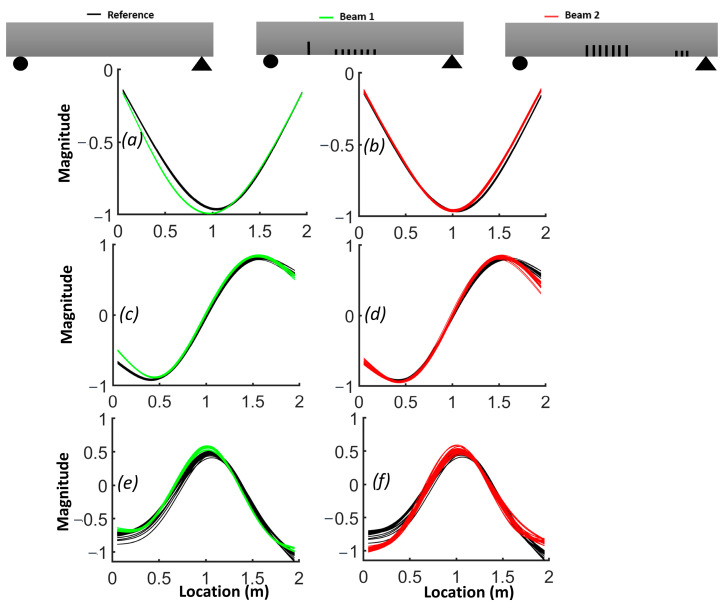
Comparison of mode shapes between beam #1, beam #2 and the reference beam: (**a**,**b**) comparison of mode 1; (**c**,**d**) comparison of mode 2; (**e**,**f**) comparison of mode 3.

**Figure 15:**
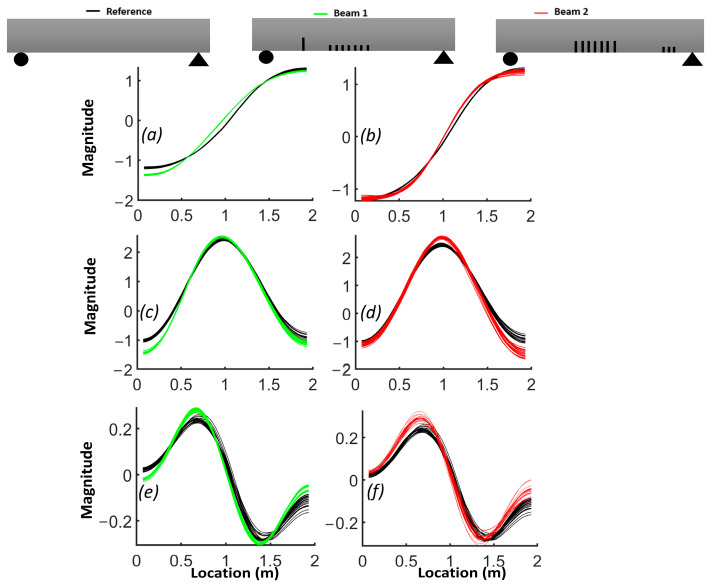
Comparison of the slope of mode shapes between beam #1, beam #2 and reference beam: (**a**,**b**) comparison of mode1; (**c**,**d**) comparison of mode 2; (**e**,**f**) comparison of mode 3.

**Figure 16:**
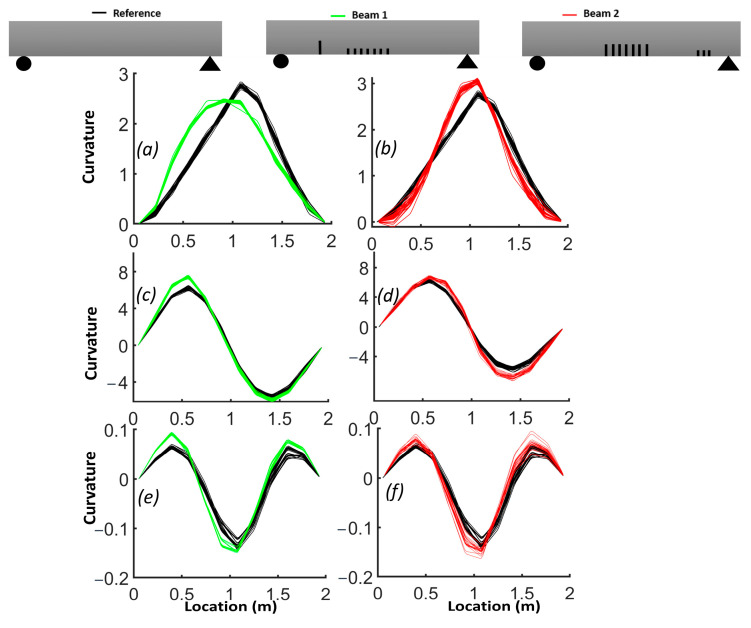
Comparison of curvature of mode shapes between beam #1 and beam #2 and reference beam: (**a**,**b**) comparison of mode 1; (**c**,**d**) comparison of mode 2; (**e**,**f**) comparison of mode 3.

Due to the sensitivity of the second derivative to noise, it is desirable to filter noise prior to its evaluation. For this purpose, each mode shape was smoothed locally with cubic smoothing splines. Figure 14 compares the first, second and third smoothed mode shapes of beam #1 and beam #2 with respect to the corresponding mode shapes of the reference beam. Notably, the first mode shapes for beams #1 and beam #2 exhibited a shift toward the side where a crack was present in the shear zone. The results for the slope of the smoothed first mode shapes show an improved correlation with the presence of cracks (Figure 15). A comparison between Figure 12 and Figure 14, as well as between Figure 13 and Figure 16, highlights the effectiveness of the proposed smoothing methods in reducing the effect of noise on the mode shapes, slopes (Figure 15) and curvature. The unsmoothed mode shapes reveal an increasing degree of variability with higher mode shapes, and this variability was further amplified for slopes and curvature, significantly impacting the detectability of defects. The results for the curvature of the smoothed mode shapes indicate enhanced detection of cracks located in the middle of the beams for the first and third mode shapes. Detection of the preformed crack of beam #1 was improved for the second mode. For beam #1, the increased curvature observed in the second and third modes on the left side was a result of a single crack located 50 cm from the left support. Additionally, the larger curvature in the middle of the third mode can be associated with damage in the middle. For beam #2, the bigger curvature values of the first and third modes in the middle section were due to damaged zones. 

Figure 16 shows a comparison of curvatures for the first three mode shapes between beam #1, beam #2 and the reference beam. The experimental curvature of mode 1 for the reference beam appears asymmetric, likely due to potential experimental uncertainties such as friction at the roller support, non-uniformly distributed fibers, etc. In the case of beam #2, the curvature of mode 1 distinctly increased with damage attributed to the cracks in the middle section of the beam. Similarly, for beam #1, the curvatures of modes 2 and 3 rose near the single crack on the left side, and for mode 1, a shift in curvature toward the side of the crack was observed without a significant increase in maximum curvature.

#### 3.3.1. Averaging DI Method

**Figure 17:**
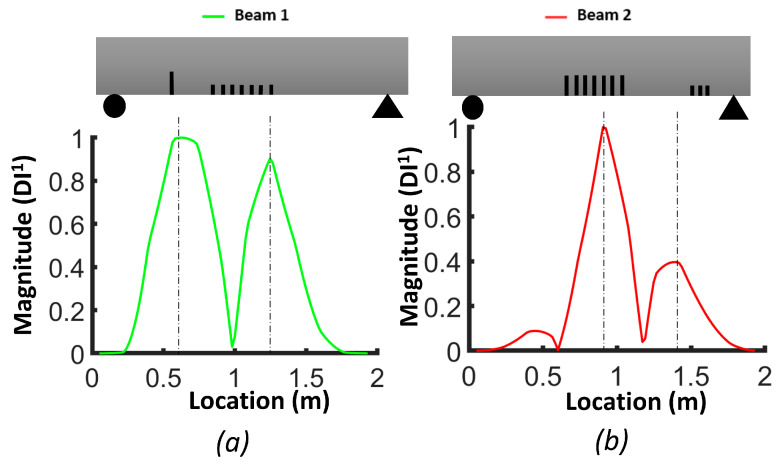
Damage index based on first mode (*DI^1^*): (**a**) beam #1; (**b**) beam #2.

**Figure 18:**
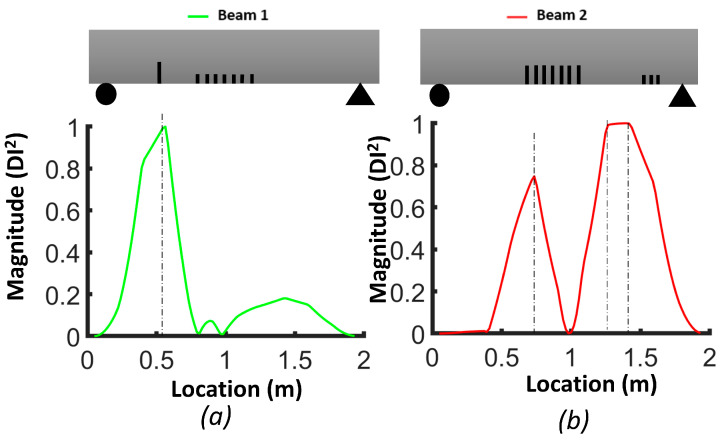
Damage index based on second mode (*DI^2^*): (**a**) beam #1; (**b**) beam #2.

**Figure 19:**
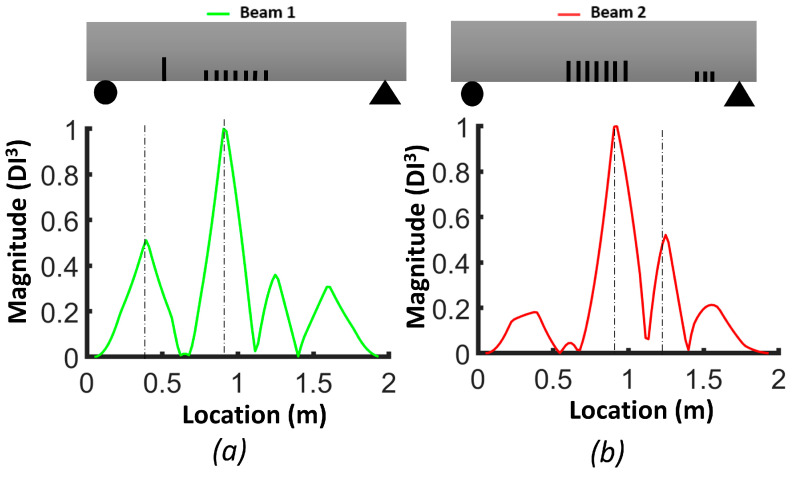
Damage index based on third mode (*DI*^3^): (**a**) beam #1; (**b**) beam #2.

**Figure 20:**
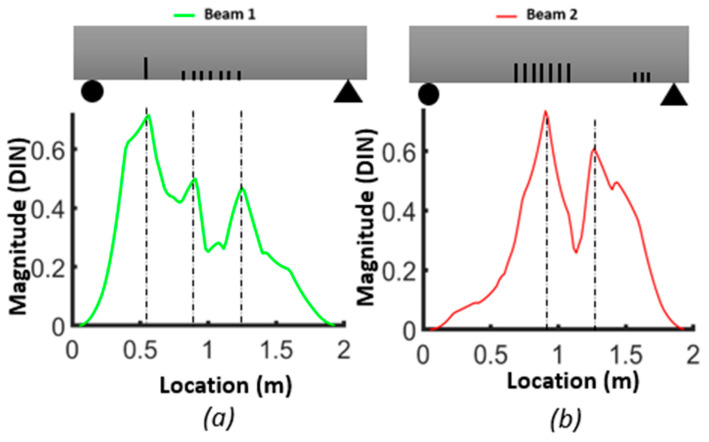
Damage index (*DIN*) considering all first three modes: (**a**) beam #1; (**b**) beam #2.

Figure 17, Figure 18 and Figure 19 show the damage index based on Equation (3) for different modes. The dashed lines delimit the region with cracks, where the *DI* reaches its maximum. For beam #1 and mode 1, the effect of the preformed crack can be observed, and its effect on displacing the curvature off-center toward the left side of the beam can also be observed. For beam #2, the position of the cracks in the mid-section was correlated with the largest differences in curvature. For the second mode of beam #1, the maximum difference corresponded to the location of the precast single crack, indicating that the discontinuity introduced during casting had a significant effect on the dynamic response of the structure. For both beams #1 and #2, the second mode was not a good indicator of damage in the mid-section due to the position of the inflection point. For beam #1, the third mode had a maximum value at x=0.92, which was the position of the middle cracks, but mode 1 did not illustrate the maximum at this position, which could be attributed to significant differences in the beams due to the non-uniformity of the beams and the effect of the single precast crack. Smaller peaks in other areas of the beams where no damage was caused indicate the lack of reliability of these criteria for damage detection. It is possible to use the curvature of higher modes, i.e., fourth and fifth modes. However, in the scope of this study, we were constrained by the availability of 12 accelerometers, and as a consequence, the analysis was limited to the first three modes. Figure 20 illustrates the *DIN* (Equation (4)), and it shows excellent results for beam #2, which had more severe cracks in the middle of the beam.

#### 3.3.2. Damage Zone Localization by WTC Method

**Figure 21:**
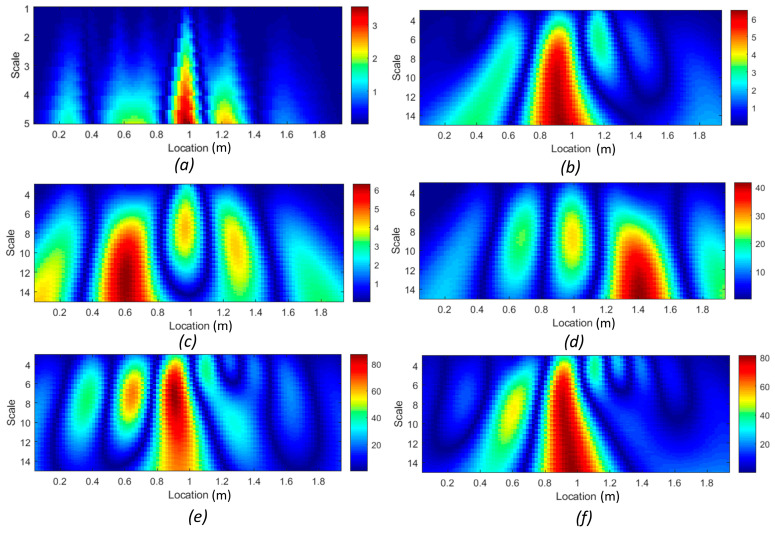
Scalogram WTC of *DI*: (**a**) *DI*^1^ of beam #1; (**b**) *DI*^1^ of beam #2; (**c**); *DI*^2^ of beam #1; (**d**) *DI*^2^ of beam #2; (**e**) *DI*^3^ of beam #1; (**f**) *DI*^3^ of beam #2.

This section presents the result of the second proposed noise reduction approach that improves the curvature-based damage detection methods. The continuous wavelet transform of the squared curvature differences for each mode was calculated separately by implementing Equation (10). Figure 21 illustrates the application of the WTC to the numerator in Equation (3) for both beam #1 and beam #2, considering different scales and modes. To address the edge effect problem, wavelet analysis was conducted on the extended signal using a zero-padding approach, ensuring that edge effects occurred outside the length of the beams. It can be concluded that, for all mode shapes, the WTC with a properly large-scale parameter could detect the positions of the cracks. From Figure 21a,b, it is evident that the WTC of the first mode shape curvature difference effectively identified the crack zone in the middle of both beam #1 and beam #2. In the case of the single crack of beam #1, the WTC of the second mode shape curvature precisely pinpointed its location at a scale greater than six (Figure 21c). However, for the shear cracks in beam #2, the WTC indicated the location of damage with a 16% error, representing the largest discrepancy for this denoising method (Figure 21d). Moreover, Figure 21f has higher wavelet scores compared to Figure 21e for the same range in the wavelet scale, suggesting a potentially greater severity of damage in the middle zone of beam #2.

#### 3.3.3. Comparison of Damage Detection Methods

**Figure 22:**
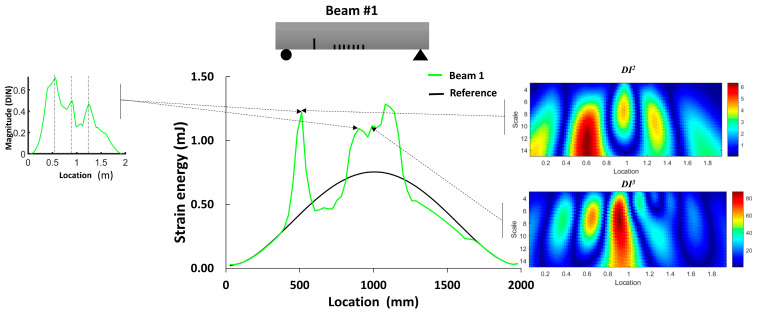
Qualitative comparison of damage detection zone for the 2 methods in comparison with the strain energy density for beam #1.

**Figure 23:**
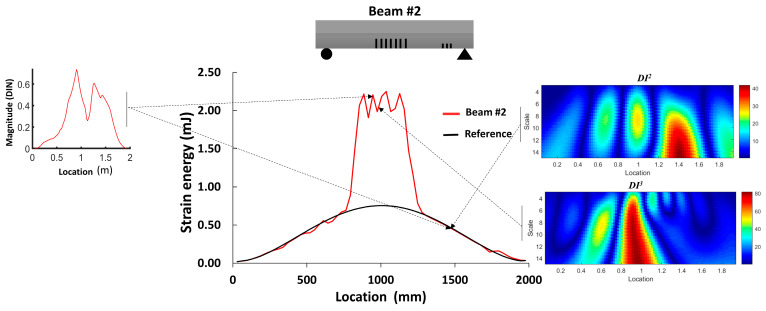
Qualitative comparison of damage detection zone for the 2 methods in comparison with the strain energy density for beam #2.

Figure 22 and Figure 23 compare the results of damage location based on the two proposed damage detection methods and their abilities to mitigate the influence of measurement noise on curvature calculations. Regarding the first method, the *DIN* could detect the middle crack in beams #1 and #2 but failed to detect the smaller cracks near the support in beam #2. In terms of multiple damage detection, the *DIN* determined the location of the start and end of the damaged zone. For cracks between them, a special threshold value should be selected.

For the second method, the strain energy was well correlated with the presence of the middle cracks and cracks near the support in beam #1 and beam #2. Moreover, the WTC method applied to the first and third absolute squared curvature differences could detect the cracks in the middle section of both beams, and its application to the second mode could detect cracks near the supports in both beams. So, the WTC-based method is more effective in identifying crack zones, particularly when the spatial scale parameter is properly selected.

In concrete structures where the likelihood of multiple spatially distributed cracks is common, it is crucial to properly select the threshold level for the *DI* to determine the extent of the damage zone. On the other hand, when using the WTC, the extent of the damage zone can be more readily determined by using a large-scale parameter.

## 4. Conclusions and Outlook

This work applies the mode shape curvature-based algorithm to detect and localize multiple damage locations in UHPFRC beams with multiple pre-damaged zones by experimental vibration measurements and FEM modeling. Based on the presented results, the following conclusions can be drawn for the two damage indices and their efficiency in detecting multiple cracks from noisy measurements.

1. Based on the measurement with 12 accelerometers with a spacing-to-span ratio of 7% (i.e., 15 cm over a span of 2 m), the Automated Frequency Domain Decomposition (AFDD) method successfully found the modal properties for the damaged UHPFRC beam, including frequencies, mode shapes and damping ratios. The average differences in the first three frequencies between the FEM and experimental results were 7.4%, 2.8% and 4.8% respectively.
2. An existing curvature damage detection method [21] with cubic smoothing splines for curve fitting and data smoothing was applied to analyze the experimental data as well as the numerical data generated by the FEM. The curvature variation in the mode shapes served as an indicator of damage location. Notably, the DIN damage index accurately identified the multiple damaged zones in agreement with the strain energy density. The accuracy of the DIN damage index in identifying the position of the cracked zone in the midspan of beam #1 and beam #2 was about 9% and 4% for the FEM-estimated acceleration data and 8% and 9% for the experimental acceleration data, respectively. The accuracy of the DIN damage index in identifying the position of the single crack in the shear zone of beam #1 was about 1% for the FEM data and 12% for the experimental data. For beam #2, the position of cracks was determined with less than 1% error for the FEM data, whereas the error increased to 20% for the experimental data.
3. The Wavelet Transform Curvature (WTC) served as an alternative approach applied to each specific Damage Index (DI) for different modes, demonstrating its efficiency in noise removal and enhanced damage detection by considering both the jerk and snap of mode shapes.

Future developments of these methods include (i) its application with other methods of measurement (e.g., laser vibrometer or experimental modal analysis with a roving hammer), (ii) applying the methods to progressively damaged beams from incremental loads and (iii) extending the methods to reference-free assessments and to existing structures.

## Figures and Tables

**Table 1:** UHPFRC composition.

Components	Density (kg/m^3^)
Premix	2195
Water	118
Premia 150	30
Steel fiber (2%)	156

**Table 2:** Mechanical properties of UHPFRC at 28 days (2% fiber) and steel fiber.

UHPFRC Properties		Fiber Properties	
Compressive strength (28 days) (GPA)	150	Diameter (mm)	0.2
Poisson’s ratio	0.2	Length (mm)	13
Young’s modulus (GPA)	56	Nominal strength (MPa)	2850
Creep ratio	1	Nominal Young’s modulus (GPa)	200
Density (kg/m^3^)	2500	Specific gravity (g/cm^3^)	7.8

**Table 3:** Natural frequencies from FE models.

Beam	f_1_ Hz	f_2_ Hz	f_3_ Hz	f_4_ Hz	f_5_ Hz
Reference	71.86	279.12	600.4	1008.6	1475.9
Beam #1	60.63 (−15.6%)	253.72 (−9.1%)	532.76 (−11.2%)	965.58 (−4.2%)	1376.8 (−6.7%)
Beam #2	52.73 (−26.6%)	264.01 (−5.4%)	507.05 (−15.5%)	1165.07 (−15.5%)	1357 (−8%)

**Table 4:** Average natural frequencies for the first five bending modes (standard deviation in parentheses).

Beam	f_1_	Diff (%)	f_2_	Diff (%)	f_3_	Diff (%)	f_4_	Diff (%)	f_5_	Diff (%)
Reference	65.90 (0)	-	271.58 (0.618)	-	550.71 (1.586)	-	1224.82 (0.35)	-	1736.40 (0.52)	-
Beam #1	65.90 (0.0023)	−0.0007	262.58 (0.696)	−3.31	545.78 (0.881)	−0.89	1211.3 (0.607)	−1.10	1626.04 (0.613)	−6.36
Beam #2	50.04 (0)	−24.07	257.81 (0.803)	−5.07	487.94 (1.514)	−11.40	1007.74 (0.630)	−17.72	1485.62 (0.691)	−14.44

**Table 5:** Average damping ratios for the first five bending modes (standard deviation in parentheses).

Beam	Mode 1	Mode 2	Mode 3	Mode 4	Mode 5
Reference	1.79 (0.0011)	3.98 (0.0024)	3.96 (0.0015)	0.88 (0.0008)	0.38 (0.0019)
Beam #1	1.70 (0.0031)	3.70 (0.0023)	3.74 (0.0008)	1.19 (0.0013)	0.72 (0.0005)
Beam #2	2.62 (0.0032)	3.70 (0.0025)	4.73 (0.0009)	1.46 (0.0008)	1.02 (0.0002)

## Data Availability

Data sharing is not applicable to this article.

## Abbreviations

*f_y_*	Yield strength of steel reinforcement	*DI*	Damage index
*ζ*	Damping ratio	*M*	Moment
*L*	Length of beam	*r*	Reference
*f*	Frequency	*d*	Damage
*ø*	Mode shape	*N*	Mode number
*Hz*	Hertz	*v*	Voltage
